# More than Just Story Telling: A Review of Biodiversity Conservation and Utilisation from Precolonial to Postcolonial Zimbabwe

**DOI:** 10.1155/2018/6214318

**Published:** 2018-08-19

**Authors:** Tanyaradzwa Chigonda

**Affiliations:** Department of Physics, Geography and Environmental Science, Great Zimbabwe University, Masvingo, Zimbabwe

## Abstract

Access to natural resources has changed over the years in Zimbabwe. At least three broad periods of biodiversity conservation, utilisation, and access can be identified in the country, namely, the precolonial, colonial, and postindependence periods. This paper reviews the relationships between human livelihoods and biodiversity conservation in the rural areas of Zimbabwe during these periods and is informed by an extensive review of the relevant literature. A combination of historical narrative, thematic, and content analysis was used in analysing the various documents into meaningful information addressing the objective of the study. Traditional societies in precolonial Zimbabwe had access to abundant natural resources. However, access to these resources was not uncontrolled, but was limited by traditional beliefs, taboos, and customs enforced through community leadership structures. The advent of colonialism in the late 19th century dispossessed indigenous African communities of natural resources through command-type conservation legislation. At independence in 1980, the new majority government sought to redress the natural resource ownership imbalances created during colonialism, culminating in some significant measure of devolution in natural resource management to local communities in the late 1980s, though such devolution has been criticised for being incomplete. An accelerated land reform exercise since the year 2000 has adversely affected biodiversity conservation activities in the country, including the conservation-related livelihood benefits derived from protected areas. The review paper highlights the need for a more complete devolution of natural resource ownership and management down to the grassroots levels in the communal areas, if social and ecological sustainability is to be fully realised in these areas. On the other hand, the disruption of conservation activities in the country due to the ill-planned accelerated land reform exercise that has demarcated land for arable farming in some of the protected areas should be held in check as a matter of urgency.

## 1. Introduction

Article 2 of the Convention on Biological Diversity (CBD) defines biodiversity as:

  The variability among living organisms from all sources, including among others, terrestrial, marine and other aquatic ecosystems and the ecological complexes of which they are part; this includes diversity within species, between species and of ecosystems [1].

Since time immemorial, biodiversity has been central to human survival [2, 3]. In prehistoric times, people more directly relied on the diversity of life in the wild for the provision of daily survival needs such as food, medicines, and shelter among others. The advent of agriculture enabled humans to domesticate wild animals and plants, necessitating a more sedentary lifestyle which, to some extent, reduced direct dependence on wild biological resources. The advent of the industrial revolution further improved agricultural production through various technologies, thereby further reducing man's direct dependence on the plant and animal resources in the wild.

However, in spite of the advances in agriculture and technology highlighted above, people around the world still rely on biological resources in the wild to meet their various needs [4]. This is particularly so in the Less Economically Developed Countries (LEDCs) where hundreds of millions of people, due to poverty, still depend on wild biodiversity for food, income, medicines, and shelter among other needs. An estimated 1.6 billion people around the world depend on forest resources for livelihoods, the majority of whom are located in the tropical regions in LEDCs [5]. Increasing populations in the poor regions of the world amid high poverty levels are further fuelling the demand for forest resources. It is, however, also important to note that, even in the developed world, forest resources are still crucial in meeting various societal needs. For example, wild plant and animal species supply genes for the improvement of cultivated and domesticated species for increasing yields, tolerances, vigour, and disease and pest resistance [6, 7]. In addition, of the 520 new drugs approved between 1983 and 1994, 39% were natural biological products or were derived from them [6].

The continued demand for natural living resources is increasingly threatening these resources with overexploitation and extinction, culminating in calls to conserve the world's biological diversity so as to ensure their sustainable use. The need for the sustainable utilisation of biodiversity has resulted in the creation of various forms of protected areas across the globe. Linkages between biodiversity conservation and livelihoods were recognised in the 1970s and early 1980s culminating in the formulation of the World Conservation Strategy, which highlighted the finiteness of natural resources and emphasised the need for ensuring their sustainable use [8, 9]. Recognition of the importance of biodiversity conservation and its linkages to global development issues was further underscored at the 1972 Stockholm Conference on the Human Environment where the inextricable link between biodiversity conservation and human development was highlighted [10]. More recently, the Sustainable Development Goals (SDGs) of the 2030 Agenda for a better future for humanity free from poverty and hunger highlight the importance of sustainably utilising natural resources towards the attainment of this ambitious agenda.

There has, however, been some fierce debate among researchers on the nature and extent of the links between biodiversity conservation and livelihoods. Scholars like Lockwood et al. [11] note that protected areas are detrimental to livelihoods through denying communities access to traditionally used resources. Other scholars [12, 13] highlight the importance of protected areas in sustaining the flow of various ecosystem services upon which humans depend for survival. Other commentators note that the impact of biodiversity conservation on local livelihoods depends on the contexts under which the protected areas are established and managed [14, 15].

Access to, and use of, natural resources has changed over the years in Zimbabwe. At least three broad periods of natural resource conservation and utilisation can be identified in the country, namely, the precolonial period, the colonial period, and the postindependence period [16, 17]. The postindependence period can, however, more conveniently be discussed as two separate periods which are the first two decades after independence (1980–2000) and the third decade starting in 2000. While these periods represent the country's political epochs, they also strongly, and differently, influenced natural resource governance and management structures. The precolonial epoch was based on the traditional leadership and their invisible fences; the colonial period involved an oppressive white-supremacist system, while the postcolonial epoch ushered in populist governance based on majority rule. This paper reviews the relationship between local livelihoods and natural resource conservation (particularly biodiversity) in the rural areas of Zimbabwe during these three periods.

Perhaps an important question to ask is why bother about the history and evolution of biodiversity conservation in relation to rural livelihoods in Zimbabwe. It is generally believed that a peep into the past is likely to enhance our understanding of current and possible future events, phenomena, and occurrences [18]. This thinking is more clearly articulated by Osakwe [19] who contends that, “To be able to appreciate fully the present, we must know something of the past.”

The aforementioned point makes a lot of sense particularly if one considers that biodiversity conservation approaches are constantly changing and evolving in order to establish their relevance at any given time in history. A historical perspective will therefore show how things have been done in the past, the reasons for such actions, and the resultant consequences. This may help in avoiding past mistakes, thereby guiding current (and future) courses of action towards better outcomes. The objective of the study is to assess the linkage between biodiversity conservation and human livelihoods in the rural areas of Zimbabwe and adopts a historical perspective covering the precolonial, colonial, and postcolonial time periods. While previous authors have written on the history of biodiversity conservation and rural livelihoods in Zimbabwe, this particular research adopts a more-than-just-story-telling perspective and attempts to add more value to these previous researches by coming up with policy implications on the biodiversity conservation livelihood nexus in Zimbabwe.

## 2. Materials and Methods

This review paper is informed by an extensive documentary review of literature on biodiversity conservation in Zimbabwe in relation to rural livelihoods using a historical perspective. The documentary review, which included relevant policies, laws, and programmes, involved careful selection of documents so as to give a balanced and accurate view regarding the evolution of biodiversity conservation in Zimbabwe from the precolonial, colonial, to the postcolonial periods and the impacts on biodiversity-dependent rural livelihoods. Government ministries and departments and organisations involved with conservation-livelihood activities in the country were among the various organisations that were approached for the collection of documentary information.

The Internet was also very crucial in accessing relevant published and unpublished secondary documents including peer-reviewed journal articles, books, and academic theses using academic literature search engines such as Google Scholar, Scopus, and Science Direct among others [20]. Various search phrases were used in searching for articles including “biodiversity conservation and utilisation in Zimbabwe,” “biodiversity conservation and livelihoods in Zimbabwe,” “biodiversity conservation policy in Zimbabwe,” “evolution of biodiversity conservation in Zimbabwe,” and “natural resource access and use in Zimbabwe.” Preliminary selection of an article for consideration was guided by the article's topic, abstract, and keywords [20, 21]. This was then followed by an in-depth reading of all initially selected articles so as to assess their relevance as information sources for the study. A total of 46 relevant documents were finally selected for the review process.

A combination of historical narrative, thematic, and content analysis was employed in reviewing the documentary sources of information gathered. This enabled the sorting of the large volumes of documentary data into focused and meaningful information useful in addressing the objective of the research. The three historical periods (precolonial, colonial, and postcolonial) naturally became the themes into which gathered documentary data were sorted and analysed, guided by the research objective.

## 3. Results and Discussion

### 3.1. Biodiversity Conservation and Use in Precolonial Zimbabwe: Pre-1890

The people who settled in what is now Zimbabwe came into the area in the later Iron Age around AD 1000 [2]. It is estimated that the area now called Zimbabwe contained a population of between 600,000 and 700,000 people before colonialism [16]. Bouchier's description of the area as “a wilderness of bush and native timber, teeming with game of every variety which found ample feeding ground in the rich valleys and grasslands that abound in all parts of the country,” indicates that the people had access to abundant natural resources [2]. However, access to these resources was not uncontrolled but was limited by some traditional beliefs, taboos, and customs [2, 17, 22, 23]. For example, some sites were believed to be hosts to some spiritual forces, and it was taboo to visit these sacred sites [23, 24]. Such sites would include mountains and forests and visiting, hunting, collection of fruits, firewood, and other natural products was prohibited. It was believed that anyone who visited such sites would temporarily get lost, disappear forever, or become insane [24]. While the sacredness of sites is highly debatable, it is, however, clear that such myths helped protect the natural environment as some areas remained intact nature conservation areas.

Traditional societies in precolonial Zimbabwe also enforced wildlife conservation by discouraging indiscriminate killing of animals and birds. It was believed that wanton killing of wildlife was punishable by the spirits and control mechanisms were found in traditional taboos, totems, and customs [22, 23]. For example, totemism was one of the observed taboos where one was not allowed to eat their totem animal [25]. Breaking such prohibitions was believed to invite illness or the loss of teeth by the offender [24]. Totemism was a very effective wildlife conservation measure against extinction because different groups of people would now eat different kinds of animals thereby avoiding the overexploitation of certain preferred species. There were also taboos forbidding the killing of young animals and females in gestation, while in some parts of the country, harvesting of premature edible caterpillars was also taboo [2]. The killing of young animals, harvesting of premature edible insects, and the exploitation of resources before certain periods of the year was believed to result in loss of eyesight for the offender [2].

There were also taboos for protecting rare or endangered animal species under immediate threat from extinction such as the python, the pangolin, and certain rare fish species [2]. For example, society believed that if one killed a python, rain would not fall, and therefore it became a protected species as killing it was now associated with the occurrence of drought [24]. The killing of such rare species could only be done with the approval of the chief [2].

There were also some traditional taboos restricting the cutting and using of certain types of vegetation. For example, indigenous fruit trees such as Muzhanje (*vapaka kirkiana*), Mutamba (*Strychnos species*), Mutohwe (*Azanza garkaena*), and Munhengeni (*ximena*), among others could not be used as firewood [22, 23]. As a way of further discouraging people from using these trees as firewood, the trees were said not to burn properly, did not last in the fire, and produced a lot of chocking smoke [24]. Such explanations were merely meant to protect these trees so as to ensure a continuous supply of fruit that were an important part of the people's diet [25]. Some trees such as Muhacha (*Parinari curatellifolia*) were nutritionally and culturally significant [22, 23]. The fruit of the tree was important for both animals and humans especially during droughts while rain-making ceremonies were also performed under it, and thus cutting it was strictly forbidden [24].

The above and other traditional taboos and customs enabled the people in precolonial Zimbabwe to live in harmony with nature by maintaining a healthy balance between them and their environment. These people were not only close to but part of nature, acknowledging that their very existence depended on it [2]. For this reason, no single major environmental challenge has been reported by any researcher to date in Zimbabwe during this period.

### 3.2. Biodiversity Conservation and Use in the Colonial Period: 1890–1980

The advent of colonialism in the last decade of the 19th century in Zimbabwe severely disrupted the harmony and close ties that had existed between the indigenous people and nature [2]. The white colonialists saw the indigenous populations as “a bunch of ignoramuses” who feared nature and thus failed to tame it for their benefit [26].

The newly established colonial administration soon introduced protective and command type natural resource and wildlife conservation legislation in order to preserve once plentiful wildlife populations which had been severely decimated by the great rinderpest epidemic of 1896-1897 combined with exploitation by slave traders, hunter explorers, prospectors, and adventurers [27, 28]. For example, the 1893 Game Law Amendment Ordinance made it illegal to sell, barter, or hawk game without a licence [2, 28]. Different licences were required for killing, catching, pursuing, hunting, or shooting game with each licence costing three pounds [2]. What this meant is that the local people could no longer hunt as in the past as they could not afford the high licence charges.

The Game Law Amendment Ordinance was replaced by the 1929 Game and Fish Preservation Act which gave the governor of colonial Zimbabwe sweeping powers to control the exploitation of wildlife [27]. The law also provided for the establishment of Wankie Game Reserve (now Hwange National Park), Vitoria Falls Reserve, Matobo (now Matoposi) National Park, and Urungwe (now Hurungwe) Game Reserve which set the foundation for the current system of protected areas in the country that now covers approximately 12% of the country's total land area [17, 27, 28]. Hunting for game was banned in the newly created protected areas and indigenous populations living adjacent to these areas could no longer hunt for sustenance as hunting became poaching [2]. The Game and Fish Preservation Act was amended in 1938 in tune with the 1933 International Convention for the Protection of the Fauna and Flora of Africa which made additional provisions to control trade in wildlife products and the movement of trophies [27].

While the above laws resulted in the recovery of wildlife populations, such population increases also threatened human settlement and commercial cattle ranching by competing for grazing and harbouring pests and diseases [27, 28]. Some cattle farmers declared that they could not continue “farming in a zoo” [29]. Consequently, large-scale hunting to eradicate large mammals for the control of the spread of tsetse fly and diseases was introduced in 1920 and continued through the 1960s [27, 28]. Approximately 680 000 wild animals were killed between 1919 and 1960 in this exercise [17].

Perhaps the most influential land use planning and administrative intervention that disposed and alienated indigenous people in colonial Zimbabwe from the natural resources they had always enjoyed was the Land Apportionment Act of 1930 [30]. The Act divided all the land into European areas, African native reserves, and other areas. The gradual implementation of the Land Apportionment Act eventually led to the emergence of a landholding structure where only 4,800 large-scale white commercial farmers occupied 11.2 million hectares of land while one million communal-area families occupied only 16.3 million hectares [2]. In addition, 74% of the native reserves or communal lands were located in the marginal agricultural areas while the white commercial farmers were mainly allocated land in the prime agricultural areas [2].

Legislative controls were also established so as to govern use of natural resources in the native reserves or communal areas. The 1928 Native Reserves Forest Produce Act restricted access to forest products for native reserve residents to own use only [23]. The sale of forest products was only allowed through the issuing of a permit. Movement of forest products between communal lands was also restricted [31]. Native reserve residents were also prohibited from exploiting protected forest areas within their lands [22, 31]. The Native Reserves Forest Produce Act also prohibited Africans from exploiting reserved tree species in their lands [31]. In addition, the Act prohibited natives from using forests within their lands where commercial licences had been granted to concessionaires [16, 22]. The Native Reserves Forest Produce Act thus made it very difficult, if not impossible, for indigenous Africans to use woodland resources in the reserves. It is important to note that most of the restricted tree species included important fruit and agro-forestry species [16]. While a strict regulatory framework for natural resource use was imposed on the African population through the Native Reserves Forest Produce Act, voluntary regulation was encouraged in the white farming areas mainly through the Forest Act of 1948 [22, 23, 31]. These acts reflected the prevailing ideology of racially determined legislative controls [31].

The period 1960 to 1980 witnessed some major changes in the perception of wildlife in colonial Zimbabwe [27]. For example, there was a growing international interest in wildlife as a source of protein in Africa which contrasted sharply with colonial policies of wasteful slaughter of large numbers of wildlife on cattle ranges and in tsetse control operations [27]. On the contrary, taking advantage of the rising international interest in African wildlife and a rising demand for wildlife products and tourism, the private sector increasingly demanded control over wildlife [27, 32]. Such mounting tensions over wildlife resulted in the reexamination of wildlife management and conservation in the country culminating in the development and promulgation of the Parks and Wildlife Act of 1975 which devolved responsibility for wildlife to the private landowner [17, 27, 33, 34]. According to [35], the granting of appropriate authority to the private sector was also intended to solve three other pressing concerns in the wildlife sector. First, there was a realisation that wildlife populations within some protected areas were increasingly becoming isolated as migration routes had been severed. Second, the transfer of wildlife responsibility would ease pressure on dwindling budgets for wildlife management. Third, wildlife was becoming more and more subject to personal interests and power struggles in the government and involving the private sector would help ease such tensions [35].

The Parks and Wildlife Act, however, did not confer ownership of wildlife on landowners. The predominant legal code in southern Africa is Roman Dutch Law where the legal status of wildlife is *res nullius*, meaning that wildlife belongs to no one but the state [27, 33]. Once an animal crossed the fence of a private landowner, it immediately ceased to be theirs. The Act therefore only gave incentives for landowners to manage and benefit from wildlife resources on their lands [27].

The Parks and Wildlife Act laid the foundation for the initial and subsequent development of the wildlife industry in Zimbabwe. According to Bond and Cumming [27], it was not until some wildlife ranches diversified into commercial safari hunting that wildlife became a viable alternative to beef production.

Unfortunately, the appropriate authority for managing and utilising wildlife conferred to private landowners through the Parks and Wildlife Act was not extended to the communal areas [16, 27]. Instead, the Act allowed for the devolution of appropriate authority for wildlife in the communal lands to a designated officer, the Secretary for Internal Affairs responsible for administering communal lands, who delegated it to the District Commissioners [16]. This meant that indigenous Africans in the communal lands remained with no access to natural resources in their lands in stark contrast to the white private landowners who could now freely utilise wildlife resources on their lands.

Perhaps, the only major attempt towards allowing indigenous Africans in the communal lands to use and benefit from natural resources during colonialism was the Wildlife Industries New Development for All (WINDFALL) project in the late 1970s [27, 34]. The primary objective of WINDFALL was to model the success registered in commercial wildlife ranching to communal areas and to reduce human-wildlife conflicts that were becoming common [17]. Under WINDFALL, meat was returned to surrounding villages from elephant culling. The experimental project failed mainly because of local community marginalisation, ambiguity, negative perceptions, and retention of revenue by government agencies including the Department of National Parks and Wildlife Management (DNPWLM) and Rural District Councils (RDCs) [17, 27].

The colonial period in Zimbabwe thus resulted in the loss of access to natural resources by indigenous African communities who had depended on these for centuries for their sustenance. These resources were now under the control of white settlers through “accumulation by displacement.” Natural resources in the white-owned areas were largely underutilised, while overcrowding in the communal areas, coupled with high poverty levels and taxation, resulted in overexploitation of resources in spite of the various resource access and use restrictions that were imposed. Colonialism also disrupted the sustainable traditional natural resource management and utilisation institutions that the indigenous Africans had developed over a long period of time. Additionally, settler actions such as the decimation of large numbers of wild animals considered as pests and the opening up of large tracts of land for agriculture, mining, and settlement set the foundation for the socioeconomic and ecological challenges the country is facing today.

### 3.3. Biodiversity Conservation and Use after Independence: 1980–Present

This section explores natural resource access and use after independence in Zimbabwe. In particular, it looks at wildlife conservation and use on private and communal lands and the impact of the fast-track land reform on biodiversity conservation and livelihoods.

#### 3.3.1. Wildlife Conservation and Use on Private Lands

The passing of the Parks and Wildlife Act of 1975, which devolved appropriate authority over wildlife to landowners, laid the foundation for private wildlife conservation in Zimbabwe. While the government remains responsible for wildlife within the Parks and Wildlife Estate, wildlife conservation has increasingly been transferred to the private sector since the 1970s through policies that encourage the devolution of authority and responsibility for wildlife to the landholder, coupled with the definition of wildlife as an economic resource [35].

Though the roots of private wildlife ranching in Zimbabwe can be traced back to the 1970s, its real growth and establishment was experienced after independence particularly in the 1980s up to the late 1990s. While security of tenure, which remained guaranteed after independence through the Lancaster House Agreement, was a necessary condition for the management of wildlife by farms in the large-scale commercial farming sector, it was not a sufficient factor for the successful establishment of the wildlife industry [27]. Changing macroeconomic conditions and the growth in live game sales and tourism in the 1990s improved the incentive structure for wildlife which saw many farms increasingly allocating resources to the management of wildlife [27, 36]. A decline in beef prices and severe droughts in the early 1990s, coupled with the collapse of the Zimbabwean dollar and a broader shift towards export-oriented agriculture, also greatly increased the popularity of game ranching [36].

In addition, a research programme in the 1980s and 1990s coordinated by the WWF on multispecies systems of animal production across the country revealed highly favourable comparisons of the wildlife industry's prospects with those of beef production [33, 36, 37]. The average financial return on investment of wildlife enterprises was 10% for Natural Regions III and IV while it exceeded 10% for Natural Region V [27, 33]. In contrast, the average return on investment to cattle was below 5% in all natural regions against a 10% level considered profitable by the survey [27]. The significant changes in land use, where ranchers destocked cattle in favour of wildlife during the period 1990 to 2000, fully substantiated the results of the WWF survey [27, 37, 38]. A further indicator of the relative viability of wildlife over livestock production systems was the formation of wildlife conservancies such as the Save Valley, Bubiana, and Chiredzi River [36, 37]. By 1994, wildlife ranching was one of the fastest growing new uses of commercial farmland in Zimbabwe, emerging into a robust and diversified wildlife sector by 1997 [27, 33, 39].

The rapid growth and establishment of the private conservation sector, particularly after independence, was an important development for the Parks and Wildlife Department for both economic and political reasons [35]. As shown earlier, it became increasingly clear in the 1980s that conservation budgets were no longer able to keep pace with spiralling conservation costs and hence, wildlife conservation on private lands presented itself as an effective cost-saving management plan. In addition, the giving away of some control over wildlife to private individuals and groups was a political move by the Parks Department to prevent the growing corrupt use of wildlife by certain elements in the Department, Ministry of Environment and Tourism and in government [35]. So, prior to 2000, there were 669 game farms and conservancies registered with the Wildlife Producers Association of Zimbabwe with a combined area of 2.5 million hectares and constituting at least 20% of the country's commercial farmland (about 5% of the country) [39].

The development of private conservancies and game ranching in the large-scale commercial farming sector was, however, criticised for perpetuating the existing unequal distribution of land and economic power [33, 35, 36]. Political and historical factors saw white commercial farmers becoming the main beneficiaries of the development of the conservancies and game ranches due to colonial policies which reserved the best land for European Areas [35]. Additionally, there was no regulatory framework for the rapidly growing conservancies, and thus, they were viewed as attempts by white commercial farmers to hide and privatise a national resource [36]. Due to the above and other reasons, some elements in government treated the transfer of appropriate authority over wildlife to private landowners with caution.

The expiry of the Lancaster House Agreement in 1990, and the subsequent enactment of the Land Acquisition Act in 1992, presented a major threat to wildlife ranches and conservancies as government could now easily and compulsorily acquire any land for resettlement [40]. In 1995, the then Minister of Environment and Tourism stated that conservancies had to be closely monitored in order to prevent them from threatening food security as they were developing in areas suitable for both commercial and subsistence crop production [35, 36]. This was followed in the same year by a similar statement from the then Deputy Minister of Lands and Water Resources pledging to curb the expansion of game farming as it threatened to swallow up the country's crop lands [35].

In response to the growing threat to their survival, game ranchers attempted various survival strategies. Recognising that failure to indigenise would ultimately threaten their long-term survival, the white commercial wildlife ranchers tried to attract black entrepreneurs into their ventures [35]. In a move to gain some political and social legitimacy, others responded with some “community trust” and “neighbour outreach schemes” through activities such as, *inter alia*, borehole drilling, school fee handouts, and access to sacred areas for the neighbouring communities [36]. However, in spite of such moves, wildlife continued to be perceived by the wider Zimbabwean society as a white-controlled area of the economy [33, 35, 36]. The neighbour outreach schemes were criticised as cosmetic attempts to maintain the status quo or as “strategic tokenism” for attracting donor funding [36].

So, prior to the fast-track land reform programme in 2000, game farms and conservancies as a conservation strategy were viewed by many in government as a hangover from the colonial period perpetuating a racially unequal distribution of land and resources. Despite the above controversies surrounding private wildlife ranching in Zimbabwe, it is clear that a switch to wildlife farming proved to be quite successful both as a profitable form of land use and as a tool for the effective conservation of wildlife resources in the country. The neighbour outreach schemes by private conservancies have also relatively contributed to livelihood enhancement for communities bordering these protected areas.

#### 3.3.2. Wildlife Conservation and Use in the Communal Farming Sector: CAMPFIRE

In parallel with the politically controversial development of game ranching on large-scale commercial farms, there have been attempts by the state, since colonial times, to disburse wildlife revenue and devolve authority to local communities in the communal areas [17, 34, 36, 41]. This eventually culminated in the now world-renowned CAMPFIRE programme explored in this section.

The concept of CAMPFIRE has its origins in the 1970s with WINDFALL that tried to emulate successes with the use of wildlife on large-scale commercial farms [31, 41, 42]. However, WINDFALL failed as it did not propose new tenure arrangements regarding the wildlife resources and shared the financial revenues from safari hunting with district authorities and not their constituent village-level communities [34]. It was a top-down programme without the participation of the people in areas with wildlife, with all decisions coming from government agencies [34, 43, 44]. CAMPFIRE was born out of the Sebungwe regional planning exercise in the early 1980s after a realisation that the development of the Sebungwe area (Binga, Gokwe and Kariba Districts) would have to be based on wildlife utilisation as much of the region is not suitable for arable agriculture [44].

The legal mechanism through which CAMPFIRE now operates was the granting of appropriate authority to District Councils through the amendment of the Parks and Wildlife Act (1975) in 1982 [34, 41, 45, 46]. This allows revenues derived from wildlife, through safari operators, hunting concessions, and trophy fees, to be accrued by the council rather than central treasury, which in turn increases incentives for councils to invest in wildlife-based revenue-earning activities. The gross wildlife revenue received by district councils is allocated to wildlife management activities, district council levies, and wildlife producer wards [45]. The revenue devolved to subdistrict levels, mostly to wards, provides the financial incentive for individuals and households to participate in the common management of wildlife [16, 42, 45]. It was hoped that devolution of appropriate authority would eventually be extended to wards and finally villages, but there are no legal mechanisms to allow this as yet [35].

The CAMPFIRE programme has realised several remarkable achievements since its inception in 1989. Economically, total annual income from safari hunting increased rapidly from US$350 000 in 1989 to US$2 million in 2001 [47]. During the same period, CAMPFIRE areas earned a total of US$20.3 million [38, 41, 47]. Through the multiplier effects, this earned the country over US$100 million [47]. A significant improvement in marketing and a steady increase in hunting quotas accounted for the impressive growth in CAMPFIRE revenue during this period [42, 47].

Another important indicator of the economic success of CAMPFIRE has been the gross annual benefit for communities in CAMPFIRE areas [27, 42]. This increased steadily from an all-time low of 38% in 1990 up to 59% in 1995 with revenue retained by councils declining remarkably [47]. However, the revenue disbursed to communities suddenly went down to less than 50% after 1996, eventually getting back to the 1990 level of 38% by 2001, with councils now retaining around 40% of CAMPFIRE revenue thereby adversely reducing benefits to the local communities [47]. In spite of the decreasing trends, the revenue that was disbursed to communities from councils was instrumental in funding various community development projects including the building of schools and clinics, the purchasing of grinding mills, and the sinking of boreholes among other development activities. Some of the revenue disbursed to communities was paid out as household dividends though, and compared to agricultural production, the cash benefits from wildlife in most wards were merely supplementary to crop and livestock production [16]. Only a few wards with low human population densities and endowed with higher wildlife populations produce significant annual household cash dividends [27, 48].

The CAMPFIRE programme has also immensely contributed to the conservation of biodiversity in Zimbabwe [42, 48, 49]. CAMPFIRE protects an area of land roughly equivalent in size to Zimbabwe's Parks and Wildlife Estate [47]. According to Duffy [35], when CAMPFIRE protected areas and ranches under wildlife are added to the Parks and Wildlife Estate, the area of land under wildlife comes up to 33%. This is apparently a major indicator of the successful impact of the CAMPFIRE programme to biodiversity conservation in Zimbabwe.

Despite its achievements, CAMPFIRE has also been criticised on a number of areas. The main criticism of CAMPFIRE has been its failure to devolve appropriate authority to subdistrict levels. The CAMPFIRE concept was founded on the principles embodied in the 1975 Parks and Wildlife Act that had devolved authority over wildlife to large-scale freehold commercial farmers [38, 47]. Through the Act, freehold farmers and ranchers were transformed into proprietary units for wildlife with the ability to make significant management decisions including the right to hunt, to allow someone else to hunt, to buy and sell game, to carry out trophy hunting, and to accrue all benefits derived from these activities [35, 47]. Similarly, CAMPFIRE founding documents aimed at an institutional change that would grant residents in communal areas territorial rights over defined tracts of land; custody and responsibility over natural resources; and the right to benefit directly from the exploitation of the natural resources on their land [38, 46]. CAMPFIRE was thus originally intended to be a rights-based approach that would give communal-area residents significant *de jure* control over their land and natural resources [47, 50] through, as [46] puts it, natural resource cooperatives or village companies.

However, the implementation of CAMPFIRE saw a number of compromises being made, with appropriate authority for the formal control over wildlife eventually being devolved to RDCs rather than to subdistrict local communities as had originally been envisaged [17, 38, 41, 51, 52]. There were no legal mechanisms to enable the devolution of appropriate authority to wards and villages as the RDC is the lowest recognisable legal entity [35, 50]. The institutional context of the country's communal lands is such that there are no community level governance institutions with rights over a defined area of land [38, 52]. The granting of appropriate authority to RDCs gives them, rather than local communities, the power to sign contracts with hunters and to accrue all the generated income. CAMPFIRE Revenue Guidelines stipulate that the RDC cannot keep more than 15% of the revenue to cover their overheads; RDCs may receive a maximum of 26% of revenue to be spent on wildlife management activities such as law enforcement and monitoring; and the CAMPFIRE Association may receive 4% of gross revenue as a levy from councils, leaving producer communities with only 55% of gross revenue [35, 47]. This has degraded the local incentive for investing in wildlife production and conservation among communal area residents considering the huge opportunity costs they incur, including damage to crops by wild animals, and has emerged as the chief critique of CAMPFIRE [38, 53]. The ultimate goal of CAMPFIRE is for producer communities to receive 80% of all the revenue generated with the RDCs retaining not more than 20% [35], and this has not been realised as yet.

The recentralisation of powers and revenue to the RDC level was also partly as a result of the Economic Structural Adjustment Programme (ESAP) which resulted in budget cuts for all government departments [35]. Diminishing budget allocations from central government resulted in RDCs increasingly becoming unwilling to devolve appropriate authority for wildlife to producer communities [35]. The granting of appropriate authority over wildlife to RDCs instead of producer communities therefore represents a major structural and implementational shortfall within the CAMPFIRE concept and is indicative of the resistance of political and economic elites to give up access and benefits that accrue from the wildlife resources [17, 54, 55]. Because of limited rights to land and natural resources, communities have little discretion to determine actual resource use and only have rights to part of the revenue generated from resource exploitation by external interests and often cannot participate in that exploitation themselves [34]. Such natural resource decentralisation reforms can best be described as “charades” due to a lack of substantive depth of institutional reform on the ground to match the rhetoric of decentralisation, devolution, and local empowerment [38, 56–58]. These limitations are recognised by some new regional initiatives such as recent tourism joint venture models being developed in South Africa that are based on enabling communities to gain equity at all levels of the game lodge tourism industry on the basis of secure rights to land on which game lodges are developed [34]. In another regional example, CBNRM in Botswana has witnessed a more complete devolution of the benefits from wildlife to community trusts which are recognised legal entities thereby giving communities greater leverage over the use of resources and the benefits derived from such use [50, 59, 60]. Namibia has also sought to extend authority over wildlife to communal lands by passing some legislative reforms in 1996 which enabled communities to form their own self-defined “conservancies” with direct proprietorship over wildlife [38].

However, the communities in which CAMPFIRE is taking place are by no means homogenous. One of the challenges to the CAMPFIRE programme has been the emergence of some elites within the local communities participating in CAMPFIRE, desiring to appropriate to themselves some of the financial and other benefits accruing to the communities. For example, chairpersons and committee members of local CAMPFIRE committees have reportedly abused accrued CAMPFIRE revenues in some areas while, in other cases, the traditional leadership has reportedly taken over control of a supposedly community project [61, 62]. In other cases, still, community political fault lines have not spared CAMPFIRE projects [60]. Such internal power struggles within CAMPFIRE participating communities have been highlighted as a red flag by opponents of complete devolution, who have taken such occurrences as important indications that subdistrict governance structures are not effective.

#### 3.3.3. The Accelerated Land Reform and Its Impact on Biodiversity Conservation and Use

Zimbabwe has undergone significant and far-reaching political, economic, ecological, and social upheavals since adopting the accelerated land reform exercise in 2000, which has seen the country descending into a state of protracted crisis [21, 62]. This has seen its relatively strong economy reduced to being one of the weakest in the region, with a once reasonably well-functioning bureaucracy weakened [62–64]. In light of the above, the fast-track land reform programme has brought with it adverse impacts on biodiversity conservation activities in the country [21, 65], and this section shall concentrate on the impacts of the accelerated land reform exercise on private conservancies and game ranches and CAMPFIRE areas.

As a recap, it is important to note that before the fast-track land reform programme in 2000, private wildlife conservation had become well established in Zimbabwe. However, as shown earlier, the state viewed the wildlife sector with suspicion and argued that wildlife production, which requires large blocks of contiguous land, was incompatible with land reform and tended to perpetuate colonial land imbalances. With the coming of the accelerated land reform in 2000, the private wildlife sector was certainly not spared. Records indicate that 655 game farms and conservancies were acquired (wholly or partially) for resettlement during the fast-track land reform period [39]. Some of the acquired game ranches and conservancies continued with wildlife activities while others were partially or wholly converted into agricultural land [27, 39]. For example, Bubiana Conservancy, measuring 84,803 ha ceded more than 17,000 ha for the AI farming model, while Bubye River Conservancy ceded 5,600 ha also for AI resettlement [39]. The Save Valley Conservancy in Chiredzi, one of the largest private conservancies in the world, also lost some of its area to resettlement. This was in line with Statutory Instrument 288 of 2000 which set out maximum permissible farm sizes per agroecological region [66].

While there is generally a dearth of information on the current status of wildlife on the game farms and conservancies, unconfirmed reports indicate that species populations declined by between 30% and 80%, mainly as a result of inadequate supplementary provisions such as water and feeds and illegal off-take and inadequate security on those acquired farms and conservancies where the wildlife land use system was maintained [39]. The UNDP estimates that about 40% of wildlife was lost as a result of change from wildlife management to agriculture [39]. Hunting and cutting down of trees by the new farmers so as to open up virgin lands for agriculture, reduce competition for grazing from wildlife, and protect livestock from predation were some of the main reasons for the decline in wildlife. The elimination of wildlife by the new settlers was also a way of protesting against the historical loss of land where every black African was stereotyped as a potential poacher and every white man a land robber, a concept that has been termed “ecoretribution” [65].

The fast-track land reform process has also had several important impacts on CAMPFIRE. Firstly, the effective destruction of institutions for the control of land and natural resources in the large-scale commercial farming sector has also been mirrored in the communal farming sector, severely undermining some of the evolving institutions for the control and management of wildlife and wildlife habitat [27]. In addition, the demand for land created by deindustrialisation and movement of labourers off large-scale commercial farms has further exacerbated the situation [27]. Resultantly, wildlife habitat is now under greater pressure than that at any time since the start of CAMPFIRE in the late 1980s [27]. Secondly, the fast-track land reform process resulted in a shift in attention towards the large-scale commercial farming sector. This has reduced the attention that is needed to resolve the ongoing land and natural resource problems within the communal areas of Zimbabwe [27].

Perhaps one of the greatest impacts on CAMPFIRE, of the fast-track land reform exercise and the ensuing political and economic turmoil, is related to the decline in tourist arrivals into the country owing to increased negative international publicity [65]. This has seen trophy hunting declining markedly in many CAMPFIRE areas thereby depriving impoverished rural communities of a significant source of income [65, 67]. It is important to note that much of the income from CAMPFIRE has been instrumental in various community development projects such as schools and clinics, in addition to being paid out as household dividends which acted as a significant add-on to other livelihood activities such as crop and livestock production [36, 65, 67]. Inflation rates reached 1700% by 2005 while record hyperinflation overtook the economy by 2008, rendering the Zimbabwean dollar virtually worthless [62]. Under such circumstances, the losses to inflation of CAMPFIRE cash benefits were massive, thereby undermining community investment projects, in addition to rendering as worthless any household cash dividends [62]. While the Reserve Bank of Zimbabwe has scrapped the Zimbabwean dollar and adopted a multicurrency system since the later part of 2009 as a way of arresting spiralling inflation, tourist arrivals have remained low due to the country's continued negative international publicity, though, of late, the situation seems to be improving gradually.

The economic decline following the fast-track land reform programme also saw many RDCs, as the appropriate authorities for wildlife management, holding on to most of the revenue generated through CAMPFIRE as they were now facing financial challenges [47, 62]. The absence of other income or taxable options has presented itself as a strong disincentive for fiscal devolution in CAMPFIRE by RDCs [62, 68]. The reduction in the flow of benefits down to subdistrict levels in turn has reduced the incentive for wildlife conservation among many CAMPFIRE communities leading to an increase in illegal off-take in many areas.

While the area under wildlife was declining in Zimbabwe after the fast-track land reform programme, particularly for private conservancies and game ranches, the opposite was taking place in other countries in the region [27]. For example, land under wildlife expanded by 40% in Namibia and by 10% in Mozambique and Zambia between 1996 and 2000 [27].

## 4. Conclusion and Recommendations

The paper has reviewed biodiversity conservation in Zimbabwe in relation to livelihoods. The review has shown that biodiversity conservation in the country went through various periods, which also affected the livelihoods of biodiversity-dependent rural communities. The precolonial period represents an era when access to natural resources by indigenous populations was unlimited, with people and their natural environment coexisting in harmony. The colonial period was characterised by the appropriation of land by white settlers, culminating in racialised natural resource ownership and utilisation and loss of livelihoods by displaced indigenous peoples. The arrival of independence witnessed a rapid growth of private wildlife conservancies and also some devolution in natural resource management to local communities from central government through the CAMPFIRE programme.

The review paper highlights some important implications that are seminal to the attainment of a more sustainable biodiversity conservation and utilisation regime in Zimbabwe. Firstly, the paper has shown that ownership of resources is key to successful biodiversity conservation. A view now commonly held within conservation circles is that, when people own natural resources, either individually or as a clearly defined community, they are more likely to sustainably utilise those resources. It was partly due to this sense of ownership that sustainable resource conservation and use was realised during the precolonial period. Additionally, private landowners during the colonial period were also able to sustainably utilise resources on their lands. This is in clear contrast with the unsustainable natural resource management situation that subsequently developed in the communal areas during and after the colonial period, where the people had been dispossessed of the resources they had traditionally owned and exploited. While some measure of devolution in the ownership and management of natural resources in the communal areas has been effected through CAMPFIRE, the need for a more complete devolution of resource ownership and management is apparent, if CAMPFIRE is to be fully sustainable both socially and ecologically. Devolution has to reach the lowest possible levels truly representing local community institutions, with communities having full ownership, access, and management of the resources and the benefits thereof. It is sad to note that other countries in the region such as Botswana, Namibia, and South Africa that came much later than Zimbabwe in CBNRM initiatives have adopted more complete devolution pathways ahead of Zimbabwe, with such initiatives characterised by higher levels of socioeconomic and ecological sustainability for the communities involved. However, such devolution should also ensure that adequate measures are taken so as to avoid the taking over of community project benefits by emerging new elites at the grassroots level.

The ill-planned fast-track land reform exercise has, apparently, been detrimental to both biodiversity conservation activities and the livelihoods of biodiversity-dependent communities in Zimbabwe. This is especially when some of the country's protected areas were wholly or partially converted into arable land, in spite of the unsuitability of such fragile and marginal areas to cultivation. Considering that such areas are only suitable for wildlife conservation, the people who have been settled in these areas for crop production should be moved to areas more suitable for arable farming [69], leaving these areas exclusively to wildlife management activities.
